# Loop-mediated isothermal amplification (LAMP): Early detection of *Toxoplasma gondii *infection in mice

**DOI:** 10.1186/1756-3305-5-2

**Published:** 2012-01-03

**Authors:** Qing-Ming Kong, Shao-Hong Lu, Qun-Bo Tong, Di Lou, Rui Chen, Bin Zheng, Takashi Kumagai, Li-Yong Wen, Nobuo Ohta, Xiao-Nong Zhou

**Affiliations:** 1 Institute of Parasitic Diseases, Zhejiang Academy of Medical Sciences, Hangzhou 310013, China; 2 Section of Environmental Parasitology, Department of International Health Development, Division of Public Health, Graduate School of Medical and Dental Sciences, Tokyo Medical and Dental University, Tokyo, Japan; 3 National Institute of Parasitic Diseases, Chinese Center for Disease Control and Prevention, Shanghai, 200025, China

## Abstract

**Background:**

Toxoplasmosis is a widespread zoonotic parasitic disease that occurs in both animals and humans. Traditional molecular assays are often difficult to perform, especially for the early diagnosis of *Toxoplasma gondii *infections. Here, we established a novel loop-mediated isothermal amplification targeting the 529 bp repeat element (*529 bp*-LAMP) to detect *T. gondii *DNA in blood samples of experimental mice infected with tachyzoites of the RH strain.

**Findings:**

The assay was performed with Bst DNA polymerase at 65°C for 1 h. The detection limit of the *529 bp-*LAMP assay was as low as 0.6 fg of *T. gondii *DNA. The sensitivity of this assay was 100 and 1000 fold higher than that of the LAMP targeting *B1 *gene (*B1*-LAMP) and nested PCR targeting 529 bp repeat element (*529 bp*-nested PCR), respectively. The specificity of the *529 bp-*LAMP assay was determined using the DNA samples of *Trypanosoma evansi, Plasmodium falciparum, Paragonimus westermani, Schistosoma japonicum, Fasciola hepatica *and *Angiostrongylus cantonensis*. No cross-reactivity with the DNA of any parasites was found. The assay was able to detect *T. gondii *DNA in all mouse blood samples at one day post infection (dpi).

**Conclusions:**

We report the following findings: (*i*) The detection limit of the *529 bp-*LAMP assay is 0.6 fg of *T. gondii *DNA; (*ii*) The assay does not involve any cross-reactivity with the DNA of other parasites; (*iii*) This is the first report on the application of the LAMP assay for early diagnosis of toxoplasmosis in blood samples from experimentally infected mice. Due to its simplicity, sensitivity and cost-effectiveness for common use, we suggest that this assay should be used as an early diagnostic tool for health control of toxoplasmosis.

## Findings

Approximately one third of the global human population is infected with *T. gondii*, including populations in Europe, South America, Africa and several Asian countries [1-3]. This parasite can cause congenital toxoplasmosis in a developing fetus and is dangerous for patients with acquired toxoplasmosis and compromised immune systems, such as patients with acquired immune deficiency syndrome (AIDS) or patients undergoing organ transplantation [4,5]. Congenital transmission of this parasite is found in a large variety of wild animal species and livestock, such as sheep, goats, pigs, and cattle [6,7]. Ingestion of infected pork is considered to be the main source of *T. gondii *infection in humans in the United States [8]. *T. gondii *is also recognized as a major cause of abortion in farm livestock such as sheep, goats, pigs, and other domestic animals [5,9].

The diagnosis of *T. gondii *infection or toxoplasmosis can be established by isolation of the parasite, histological examination, serological tests, or polymerase chain reaction (PCR). Biological diagnosis classically relies upon serological examination and direct detection of the parasite by inoculation of laboratory animals. A serological assay is considered the most challenging procedure because specific antibodies may not be present in the early stages of infection, especially in immune deficient or pregnant patients [10]. Nested PCR, real-time PCR and LAMP assays have been used to detect *T. gondii *DNA in animal materials [11], water [12], soil [13], and clinical specimens [14]. In the past decade, the use of PCR has resulted in a significant improvement in both the prenatal diagnosis of congenital toxoplasmosis and the detection of acute disease in immunocompromised patients. Among these PCR techniques, nested PCR, followed by hybridization, has been reported to be the most sensitive assay for detection [15]. Real-Time PCR to detect toxoplasmosis not only can quantify *T. gondii *in biological samples but also has superior sensitivity over nested PCR assays [16,17]. Despite these advances, diagnosis of *T. gondii *infection remains unsatisfactory because PCR-toxoplasma assays have not yet attained a sufficient level of sensitivity, and are limited due to expensive equipment and long reaction time periods.

LAMP is one of the nucleic acid amplifications tests used in various fields, including infection diagnosis to identify organisms. This assay uses a DNA polymerase called Bst polymerase, which has displacement activity and a set of four specially designed primers that recognize a total of six distinct sequences of the target DNA [18]. It has been used to perform highly specific and sensitive amplifications of DNA to detect pathogens including viruses, bacteria, protozoa, and fungi. Recently, this technique has proven to be very useful in the diagnosis of parasitic infections, such as malaria, trypanosomiasis, dirofilariasis, and babesiosis [19-22]. Rapid detection of *T. gondii *in water samples by LAMP was first described in a study by Sotiriadou *et al*. [23]. Thereafter, the LAMP assay was developed and evaluated for the detection of *T. gondii *infection from the lymph nodes of pigs [24], various organs harvested from mice [25], and blood samples from patients [26].

In the present study, we developed a LAMP assay targeting the 529 bp repeat element for the detection of *T. gondii*. Furthermore, we evaluated the detection sensitivity of *T. gondii *LAMP in comparison with conventional nested PCR. This is the first report in which the LAMP assay has been used for early diagnosis of active toxoplasmosis in mouse blood samples.

### Development and optimization of the 529 bp-LAMP assay

**Figure 1:**
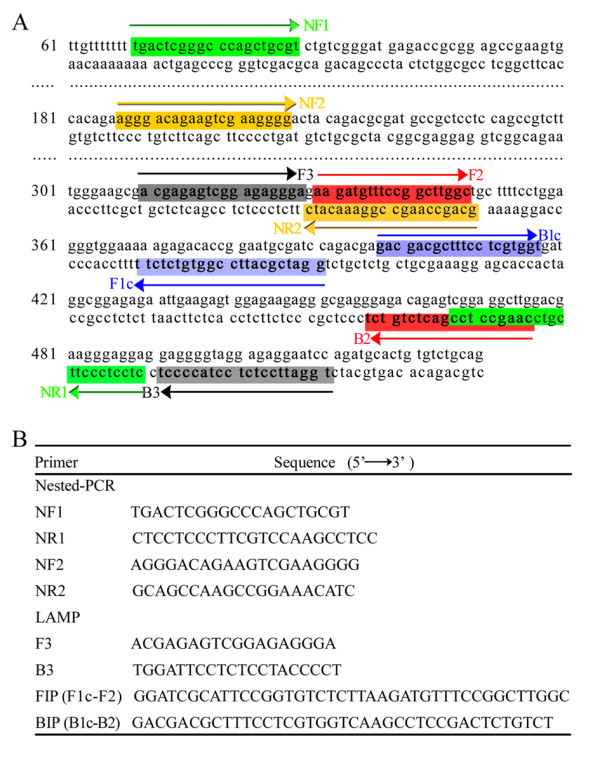
**Location and sequence of LAMP primer sets targeting the *T. gondii *529 bp repeat element**. (A) Partial sequence of *T. gondii *529 bp repeat element and the location of four primers: F3, B3, FIP (F1c-F2) and BIP (B1c-B2). Arrows indicate the direction of extension; numbers on the left indicate the nucleotide position. (B) Sequence of primers for Nested-PCR LAMP reaction.

The choice of gene sequence is critical when establishing a diagnostic method for molecular tests. Multicopy sequences specific for *T. gondii*, such as the *B1 *gene or the 529 bp sequence used in this study, are especially useful in molecular tests [27]. For the detection of *T. gondii*, the sequence most frequently used is the *B1 *gene, first identified in 1989 by Burg *et al*. [28], of which there are 35 copies in the genome. The newly described 529 bp repeat element is repeated more than 300-fold in the genome of *T. gondii*. On the basis of the sequence for the 529 bp repeat element in GenBank (AF146527.1), one primer set for use in the LAMP assay and a second set for the nested PCR were designed. The location and sequence of each primer targeting this repeat element for *T. gondii *detection are shown in Figure 1.

**Figure 2:**
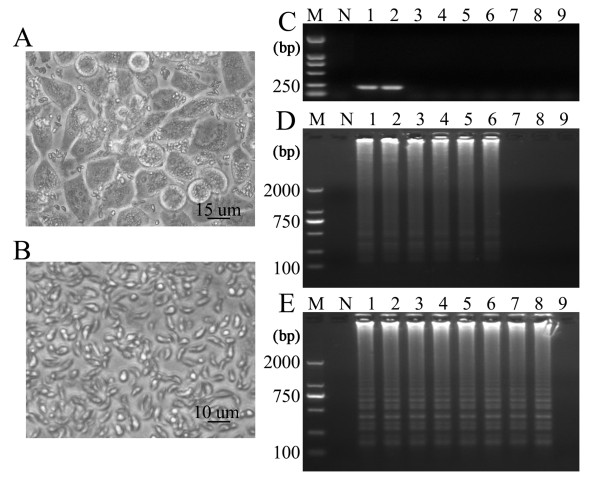
**Sensitivity of the LAMP assay and nested PCR method**. LAMP and nested PCR assays were carried out using the DNA extracted from purified *T. gondii *tachyzoite (RH strain). (A) HeLa cell monolayer infected with *T. gondii *tachyzoites in DMEM supplemented with 2% fetal bovine serum. (B) Purified tachyzoites by Percoll density gradients centrifugation. (C) *529 bp*-nested PCR reaction with *T. gondii *DNA. (D) *B1*-LAMP reaction with *T. gondii *DNA. (E) *529 bp-*LAMP reaction with *T. gondii *DNA. Lanes 1 **- **9 represent 6 ng, 600 pg, 60 pg, 6 pg, 600 fg, 60 fg, 6 fg, 0.6 fg and 0.06 fg of DNA, respectively; lane M represents a 2,000 bp DNA ladder; lane N represents a negative control.

The procedures for the LAMP and nested PCR methods were carried out according to the description by Lau *et al*. [26]. The *529 bp-*LAMP assay was performed using the Loopamp DNA amplification kit (Eiken Chemical Co. Ltd., Tokyo, Japan). In brief, the assay was performed with the following optimized reaction mixture: 25 μl of a mixture containing 12.5 μl of 2 × reaction mix buffer, 1 μl of the extracted DNA of *T. gondii *RH strain, 40 pmol (each) of primers FIP and BIP, 5 pmol of primers B3 and F3, and 1 μl of Bst DNA polymerase. To determine the optimal conditions for sensitivity and selectivity, the LAMP reactions were performed at a range of temperatures (61, 63, 65, 67 and 69°C) for different time periods (40, 50, 60, 70 and 90 min). The best result was obtained when the reaction temperature was maintained at 65°C for 60 min (Data not shown). All positive LAMP reactions produced a typical ladder of multiple bands on the 1.5% agarose gel stained with GelRed™ (Biotium Inc.) (Figure 2E), which indicated the production of stem-loop DNA with inverted repeats of the target sequence. Furthermore, positive reactions turned green on addition of SYBR Green I to reaction tubes, while the tubes showing negative reactions remained orange. The first round of *529 bp*-nested PCR amplification contained 10 μl 2 × Taq PCR MasterMix (0.1 U Taq Polymerase, 500 μM dNTP each, 20 mM Tris-HCl, pH 8.3, 100 mM KCl, 3 mM MgCl_2_), 0.3 μl of the 5 μM primers NF1 and NR1 (Figure 1), 1 μl of extracted DNA and 8.4 μl ddH_2_O. Reactions were cycled 30 times by denaturation at 94°C for 1 min, followed by annealing at 60°C for 30 s and a final extension step at 72°C for 40 s. The first round product was diluted 1:100. The second round of PCR mixtures contained 2 μl diluted product, 10 μl 2 × Taq PCR MasterMix, 1 μl of each 5 μM primer NF2 and NR2, and 6 μl ddH_2_O. The second round PCR was cycled 35 times by denaturation at 94°C for 10 s, followed by annealing at 50°C for 15 s and a final extension step at 72°C for 20 s. The PCR products of 164 bp for the positive reaction appeared on the 1.5% agarose gel stained with GelRed™ (Biotium, Inc.) (Figure 2C).

### LAMP sensitivity and specificity

*T. gondii *tachyzoites (RH strain) were propagated *in vitro *under standard procedures by serial passages in HeLa cell monolayer in Dulbecco's modified Eagle medium (DMEM, Invitrogen) supplemented with 2% fetal bovine serum at 37°C under 5% CO_2 _(Figure 2A). Tachyzoites were collected by scraping the cell monolayer and washing with cold phosphate-buffered saline (PBS). The final pellet was resuspended in cold PBS and passed three times through a 30-gauge needle syringe. Clarification and all subsequent centrifugations were performed at 4°C. The parasites were then diluted with 1.0 ml PBS and centrifuged to equilibrium in 13 ml non-linear 10% - 50% Percoll density gradients at 2,500 × *g *for 20 min. Purified parasites were diluted with PBS and pelleted by sedimentation at 2,500 × *g *for 20 min to remove the Percoll. The purity of parasites was confirmed microscopically to ensure that the tachyzoites had normal morphology and to exclude the possible inclusions of other cellular organelles and debris (Figure 2B). Tachyzoite genomic DNA extracted with DNeasy Blood & Tissue Kit (QIAGEN, Maryland, USA) was used in concentrations ranging from 6 ng to 0.06 fg. Three replicate assays showed high reproducibility of the LAMP and nested PCR. The DNA extraction procedure did not affect the sensitivity of the LAMP and PCR.

The sensitivity of the *529 bp-*LAMP assay was determined and compared to the results of the *529 bp*-nested PCR and the *B1*-LAMP. The LAMP products in positive reaction tubes were visually detectable on addition of SYBR Green I to the reaction tube. On gel electrophoresis, the amplified products showed ladder-like patterns. The detection limit of the *529 bp-*LAMP assay was 0.6 fg of *T. gondii *DNA (Figure 2E). In contrast, the detection limit of *B1*-LAMP was 60 fg of the DNA template (Figure 2D) and the limit of the *529 bp*-nested PCR was 600 fg of the DNA template (Figure 2C). Therefore, it appears that the sensitivity of the *529 bp-*LAMP assay was 100 fold higher than that of the *B1*-LAMP assay and was 1000 fold higher than that of the *529 bp-*nested PCR. These results demonstrate that the primers based on the 529 bp repeat element are more suitable than those based on the *B1 *gene. This supported by the finding that the sensitivity of the quantitative LightCycler PCR assay targeting the 529 bp repeat element of *T. gondii *is about 10 to 100-fold higher than that targeting the *B1 *gene [29]. The sensitivity of LAMP assay is higher than that of the conventional PCR for detection of protozoan parasites such as *Babesia *spp., *Theileria *spp., *Trypanosoma *spp., which has been reported previously [30-32].

**Figure 3:**
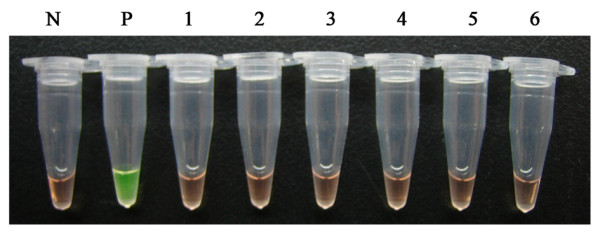
**Specificity of the LAMP assay**. Detection of DNA samples of *T. gondii *and other parasites by using the LAMP assay. Tubes of the LAMP reactions were visually inspected. Positive reactions turned green after the addition of SYBR Green I. Tube N represents the negative control; tube P represents the positive control of *T. gondii*. Tubes 1 - 6 represent *Trypanosoma evansi, Plasmodium falciparum, Paragonimus westermani, Schistosoma japonicum, Fasciola hepatica *and *Angiostrongylus cantonensis*, respectively.

The specificity of the LAMP assay was tested by using the DNA samples of other parasites. No amplification was observed in the DNA samples of *Trypanosoma evansi, Plasmodium falciparum, Paragonimus westermani, Schistosoma japonicum, Fasciola hepatica *and *Angiostrongylus cantonensis *(Figure 3), which proved that the LAMP primers are highly specific for the detection of *T. gondii*.

### Application of LAMP to blood samples from infected mice

Six-week old BALB/c mice (Purchased from the Zhejiang Provincial Experimental Animal Center, China) were infected by intraperitoneal injection of 20 virulent tachyzoites of the *T. gondii *RH strain (Stored in our laboratory). Blood samples were collected at 1, 3, and 5 dpi from the vena orbitalis posterior plexus blood. The DNA was extracted from blood samples of infected mice by using DNeasy Blood & Tissue Kit (QIAGEN, Maryland, USA) according to the manufacturer's instructions. The use of animals was approved by the Institutional Animal Care and Use Committee (IACUC) of Zhejiang Academy of Medical sciences. The purified DNA from 50 μl blood samples was dissolved in 30 μl of double-distilled water and 1 μl of the resulting supernatant was used as the template for the subsequent detection. The DNA extracted from the blood samples of the uninfected mice was used as negative control. In order to exclude the presence of inhibitors in blood samples, we selected a pair of PCR primer

(F: 5'-TCAAGAACGAAAGTCGGAGT-3';

R: 5'-GGACATCTAAGGGCATCACA-3') to amplify a mouse 18s rRNA fragment (GenBank Accession No. NR_003278.2), with an amplified zone of the target is 489 bp [33]. We also chose mouse 18s rRNA as an internal control target. The primers for internal control are as follows,

F3: 5'-GAATCAGGGTTCGATTCCGG-3';

B3: 5'-GAATTACCGCGGCTGCTG-3';

FIP: 5'-AGTGGGTAATTTGCGCGCCTGAGAGGGAGCCTGAGAAACG-3';

BIP: 5'-CAGGACTCTTTCGAGGCCCTGTGCCCTCCAATGGATCCTC-3'.

**Figure 4:**
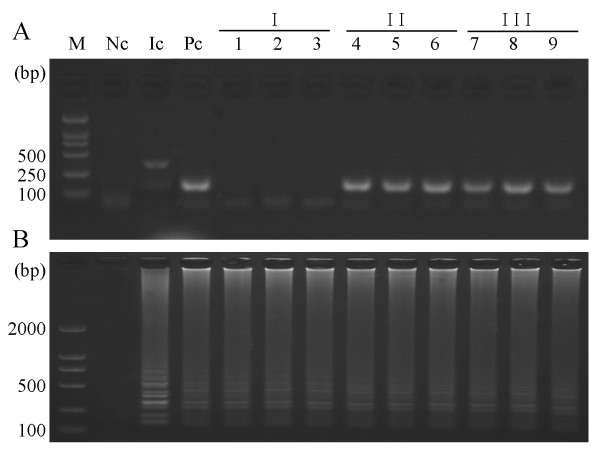
**Analysis results of 9 blood samples from *T. gondii *infected mice**. (A) *529 bp-*nested PCR; (B) *529 bp-*LAMP. I, II, III represent three groups of BALB/c mice (n = 3 mice/group) that were infected by intraperitoneal injection with 20 virulent tachyzoites of *T. gondii *RH strain. Lane M represents 2,000 bp DNA ladder; lane Nc represents negative control; lane Ic represents internal control; lane Pc represents positive control; lanes 1-9 represent sample ID. Blood samples from mice were collected at 1, 3, and 5 days post injection.

Analysis of the nine blood samples by the *529 bp-*LAMP assay and *529 bp*-nested PCR revealed some differences in the sensitivity of both the assays. The *529 bp-*LAMP assay showed positive results for all blood samples from infected mice. The nested PCR assay showed DNA fragments of appropriate sizes from six blood samples (Figure 4). The negative control of uninfected mouse did not show amplification of DNA fragments by LAMP and nested PCR. Internal control reaction was visually observed which confirmed that there were no inhibitors in blood samples. In mouse inoculated with tachyzoites of RH strain, the parasites were first detected by the nested PCR assay at 3 dpi, while the earliest detection of parasite DNA by *529 bp-*LAMP assay was at 1 dpi, which demonstrated that the LAMP assay was effective for an earlier diagnosis. In a recent report, the *B1*-based LAMP assay had a higher sensitivity (80%) than nested PCR (62.5%) in diagnosing toxoplasmosis in human blood samples [26]. To analyze the diagnostic sensitivity and specificity of the LAMP assay for detection of *T. gondii *in blood samples, we tested, through LAMP assay and nested PCR, 28 blood samples obtained from nine infected mice with 20 virulent tachyzoites of the *T. gondii *RH strain and 19 normal mice. Six out of 28 blood samples were nested PCR-positive and nine out of 28 blood samples were LAMP-positive (Data not shown). The LAMP in this study revealed a sensitivity/specificity of 100% and a higher sensitivity than the nested PCR assays. These results showed that this assay should be considered as an early diagnostic tool for toxoplasmosis in mouse blood samples. They also demonstrated that the LAMP assay has a higher sensitivity than nested PCR for detection of *T. gondii *in blood samples.

The *529 bp-*LAMP assay was also applied to various organ samples including brain, heart, liver, spleen, kidney and lung from experimentally infected mouse. The assay showed positive results for heart samples at 1 dpi, for kidney samples at 3 dpi and for all organ samples except the brain at 5 dpi (Data not shown). Kaneko *et al*. also reported that the specificity and sensitivity of LAMP detection does not seem to be impaired by sample type, including plasma, serum, PBS, saline, urine, aqueous humor and vitreous substances. The tolerance of LAMP for biological substances was very high [34,35]. Another advantage of the LAMP assay is that the requirements for LAMP are relatively simple, and that it does not require high technical skills or sophisticated equipments. However, there is a high risk of aerosol contamination due to the large amount of LAMP products. To reduce the risk of contamination, the LAMP partition in the laboratory, such as solution preparing partition, sample treatment partition, and gel electrophoresis detection partition, should be carried out in separate areas. Gloves should be changed regularly and sterile pipetting techniques should be applied during the entire LAMP experiment.

Here, we report a LAMP assay that specifically targets the 529 bp repeat element for the detection of *T. gondii *in mouse blood samples. We were able to demonstrate the successful amplification of *T. gondii *DNA at 1 dpi within 1 h at 65°C using the LAMP assay. On the basis of these results, the LAMP assay can be considered one of the most accurate molecular assays because it is a specific, sensitive, and rapid diagnostic tool for the early detection of *Toxoplasma *in blood samples.

## Competing interests

The authors declare that they have no competing interests.

## Authors' contributions

KQM and TQB performed the main experiments and data analysis. KQM drafted the manuscript. LD, ZB and TK contributed to the initial phase of the experiments and assisted in the propagation of *T. gondii *tachyzoites. CR, WLY and NO helped conceive the research. LSH and ZXN created the detailed experimental design. All authors read and approved the final manuscript.
